# Sarcoidosis-lymphoma syndrome with portal hypertension: diagnostic clues and approach

**DOI:** 10.1016/j.radcr.2021.05.045

**Published:** 2021-06-12

**Authors:** Fumio Chikamori, Kenji Yorita, Tadashi Yoshino, Satoshi Ito, Miki Mizobuchi, Koji Ueta, Kai Mizobuchi, Shigeto Shimizu, Kazumasa Nanjo, Sawaka Yukishige, Jun Iwabu, Hisashi Matsuoka, Norihiro Hokimoto, Hiromichi Yamai, Kazuhisa Onishi, Nobuyuki Tanida, Niranjan Sharma

**Affiliations:** a Department of Surgery, Japanese Red Cross Kochi Hospital, Kochi, Japan; b Department of Diagnostic Pathology, Japanese Red Cross Kochi Hospital, Kochi, Japan; c Department of Pathology, Okayama University Graduate School of Medicine, Dentistry and Pharmaceutical Sciences, Kita-ku, Okayama, Japan; d Department of Radiology, Japanese Red Cross Kochi Hospital, Kochi, Japan; e Department of Internal Medicine, Japanese Red Cross Kochi Hospital, Kochi, Japan; f Department of Surgery, Adv Train Gastroint & Organ Transp Surgery, Dunedin, New Zealand

**Keywords:** Sarcoidosis-lymphoma syndrome, Portal hypertension, Laparoscopic liver biopsy, PET/CT, Hepatic sarcoidosis, Primary hepatic Hodgkin lymphoma

## Abstract

Sarcoidosis-lymphoma syndrome associated with portal hypertension is very rare. A 68-year-old female presented with a 5 kg weight loss in 6 months. Soluble interleukin-2 receptor activity was increased and total platelet count was decreased. Contrast-enhanced computed tomography showed the presence of hepatosplenomegaly and a 3 cm-sized tumor in segment 3 of the liver. The hepatic venous catheterization showed mild portal hypertension. On fluorodeoxyglucose-positron emission tomography/computed tomography, progressive malignant lymphoma was suspected. However, bone marrow biopsy showed multiple noncaseating granulomas. A laparoscopic liver biopsy revealed that the liver tumor had features of Hodgkin lymphoma. There were multiple noncaseating epithelioid granulomas in the portal tracts of the liver. Splenectomy for splenomegaly and partial hepatectomy for the liver tumor were performed. Pathological examination of the resected specimens revealed multiple noncaseating epithelioid granulomas in the liver and spleen. Histopathology of the liver tumor confirmed classic Hodgkin lymphoma with mixed cellularity. We conclude that hepatic venous catheterization, positron emission tomography/computed tomography, and pathological examinations of bone marrow, liver, and spleen are crucial for the diagnosis of sarcoidosis-lymphoma syndrome associated with portal hypertension.

## Introduction

Sarcoidosis-lymphoma syndrome is a rare condition. It was described as coexistent sarcoidosis and malignant lymphoproliferative disease [1], [2], [3]. Definitive diagnosis of sarcoidosis-lymphoma syndrome by clinical and radiological findings is difficult. An optimal diagnostic approach has not been established [4], [5], [6]. At the same time, portal hypertension secondary to hepatic sarcoidosis is also infrequent [7], [8], [9]. We encountered a case of hepatosplenomegaly with liver tumor and hypersplenism. We report the importance of a multidisciplinary approach in the diagnosis of sarcoidosis-lymphoma syndrome with portal hypertension.

## Case report

A 68-year-old female attended our hospital for a weight loss of 5 kg in half a year. Anamnesis revealed a history of breast cancer for which the patient received surgery 2 years ago. At that time, splenomegaly and thrombocytopenia were noted. However, they were left unattended. She had no history of alcohol consumption and smoking. At the time of outpatient consultation, physical examination revealed a conscious patient with correct respiratory and hemodynamic parameters. The rest was unremarkable.

Laboratory tests revealed hemoglobin 12.9 g/dL (normal range, 11.0-14.6); total leukocyte count 3320 /µL (3500 - 8000); total platelet count 6.8 × 10^4^ /µL (12.3 – 33.1 × 10^4^); total bilirubin 1.5 mg/dL (0.3 – 1.3); albumin 4.5 g/dL (3.8-5.0); prothrombin time 87.4% (70 - 130); C-reactive protein 0.16 mg/dL (<0.16); carcinoembryonic antigen 1.9 ng/mL (0-5.0); α-Fetoprotein (AFP) 1.9 ng/mL (0-20); protein induced by vitamin K absence or antagonist-II (PIVKA-Ⅱ) 39 mAU/mL (<40); soluble interleukin-2 receptor (sIL-2R) 3365 U/mL (122-496); Mac-2 binding protein glycosylated isomers (M2BPGi) 6.29 COI (2+) (<1.00); the retention rate of indocyanine green at 15 minutes (ICG15) 32 % (<10). Antimitochondria antibody and antinuclear antibody were negative. Hepatitis B surface antigen and hepatitis C virus antibody were negative. The Child-Pugh score was 5 and the class was A. From these data, non-alcoholic steatohepatitis (NASH)-related liver fibrosis with thrombocytopenia and lymphoproliferative disease were suspected.

Abdominal ultrasonography showed the presence of hepatosplenomegaly, 3 -cm-sized homogeneous hypoechoic tumor with irregular margin in segment 3 of the liver and gallbladder stones. The mean blood flow velocity and cross-sectional area of the splenic vein were 21.5 cm/sec and 0.79 cm^2^, respectively. The splenic venous flow volume was 1013 mL/min and showed a marked increase. The mean blood flow velocity, cross-sectional area, flow volume, and congestion index of the portal vein were 35.5 cm/sec, 0.90 cm^2^, 1913 mL/min and 0.025 cm*sec, respectively.

**Fig. 1:**
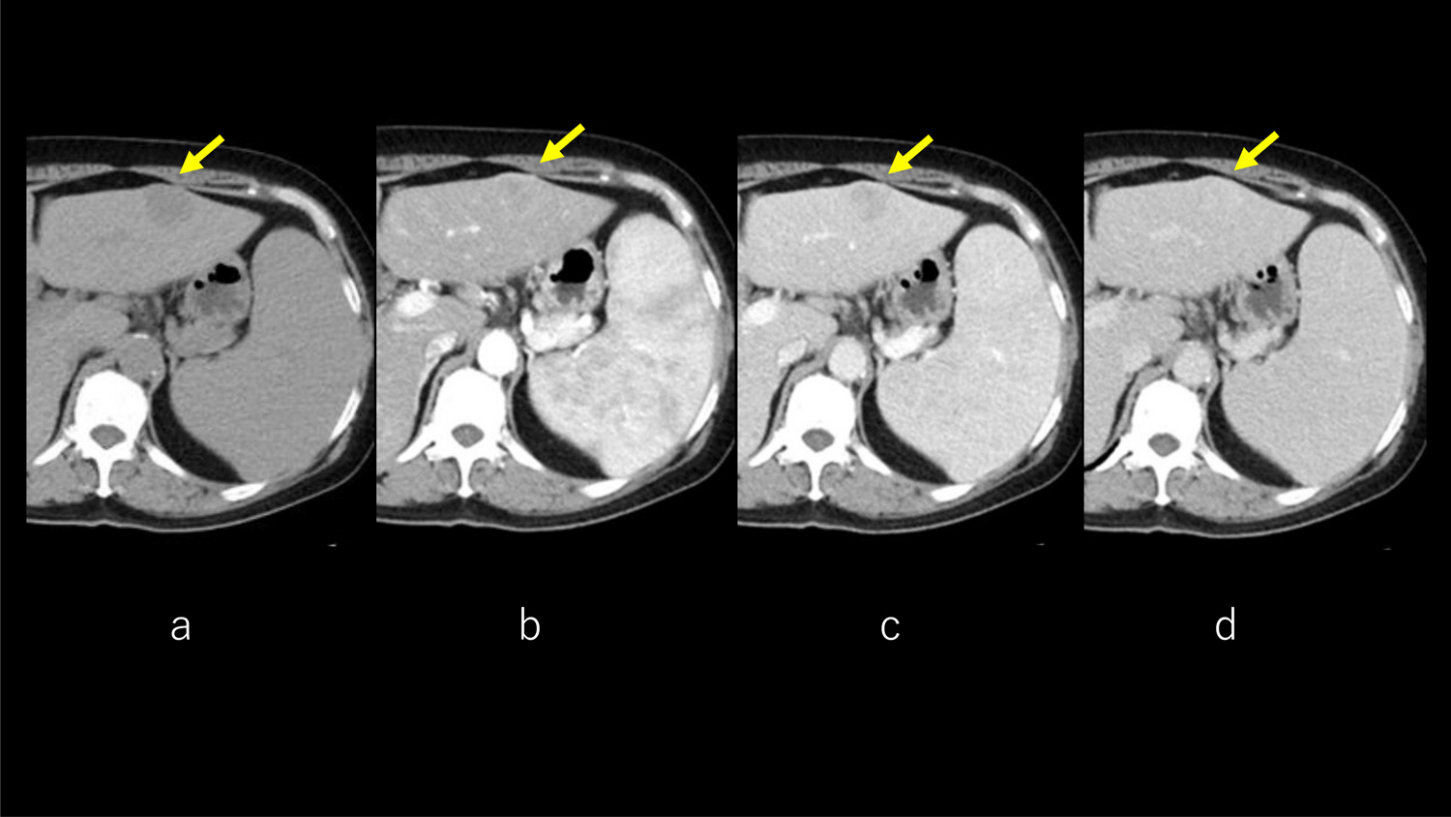
CT. Abdominal plain CT shows solitary, hypoattenuating tumor (arrow) in the left lobe **(A)**. The arterial phase of dynamic CT shows tumor enhancement with central part sparing **(B)**. The tumor is demonstrated clearly in the portal phase **(C)**. The equilibrium phase of dynamic CT shows the lesion with persistent enhancement **(D)**. CT, computed tomography.

**Fig. 2:**
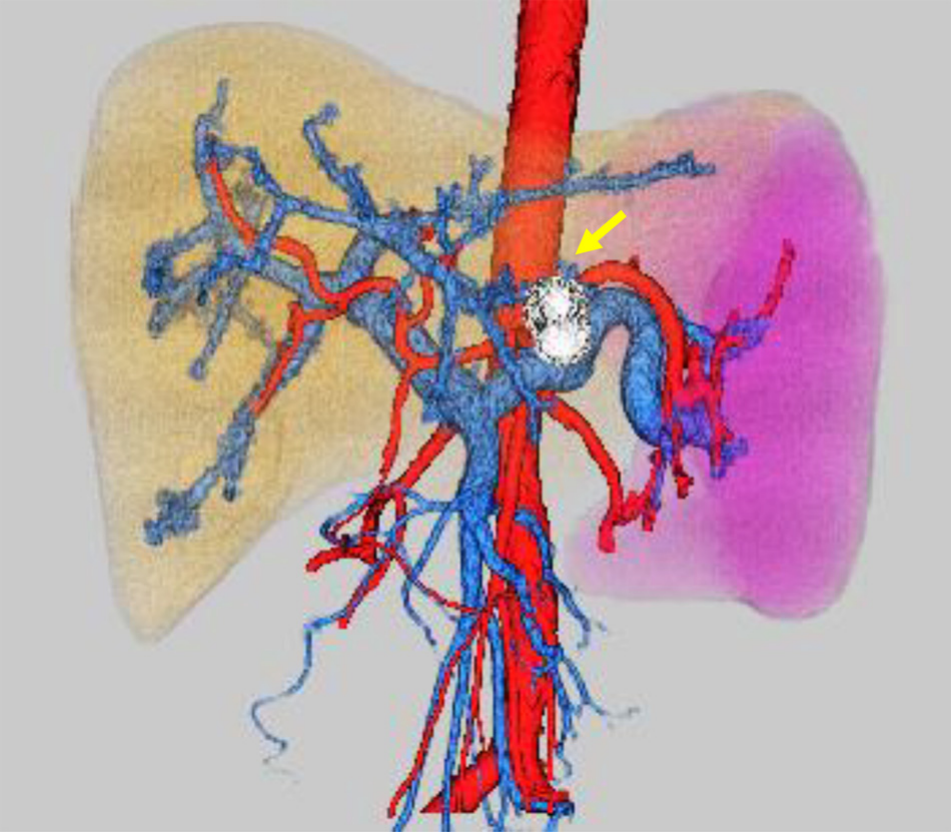
3D-CT reconstruction image. 3D-CT reconstruction image shows hepatosplenomegaly and increased splenic vein diameter. The spleen volume is 1011 mL and the liver volume is 2461 mL, respectively. Spleen/liver volume ratio is 0.41. No development of portal collaterals is observed. The tumor (arrow) is located in segment 3. CT, computed tomography.

Abdominal plain CT showed a solitary, hypoattenuating tumor in the left lobe. The arterial phase of dynamic CT showed tumor enhancement with central part sparing. The tumor was demonstrated clearly in the portal phase. The equilibrium phase of dynamic CT showed the lesion showed persistent enhancement (Fig. 1A–D). 3D-CT reconstruction image demonstrated hepatosplenomegaly and increased splenic vein diameter. The spleen volume and spleen volume/body surface area were 1011 mL and 623 mL/m^2^, the liver volume (LV) and LV/body surface area were 2461 mL and 1517 mL/m^2^, respectively. Spleen/LV ratio [10] was 0.41. No development of portal collaterals was observed (Fig. 2).

**Fig. 3:**
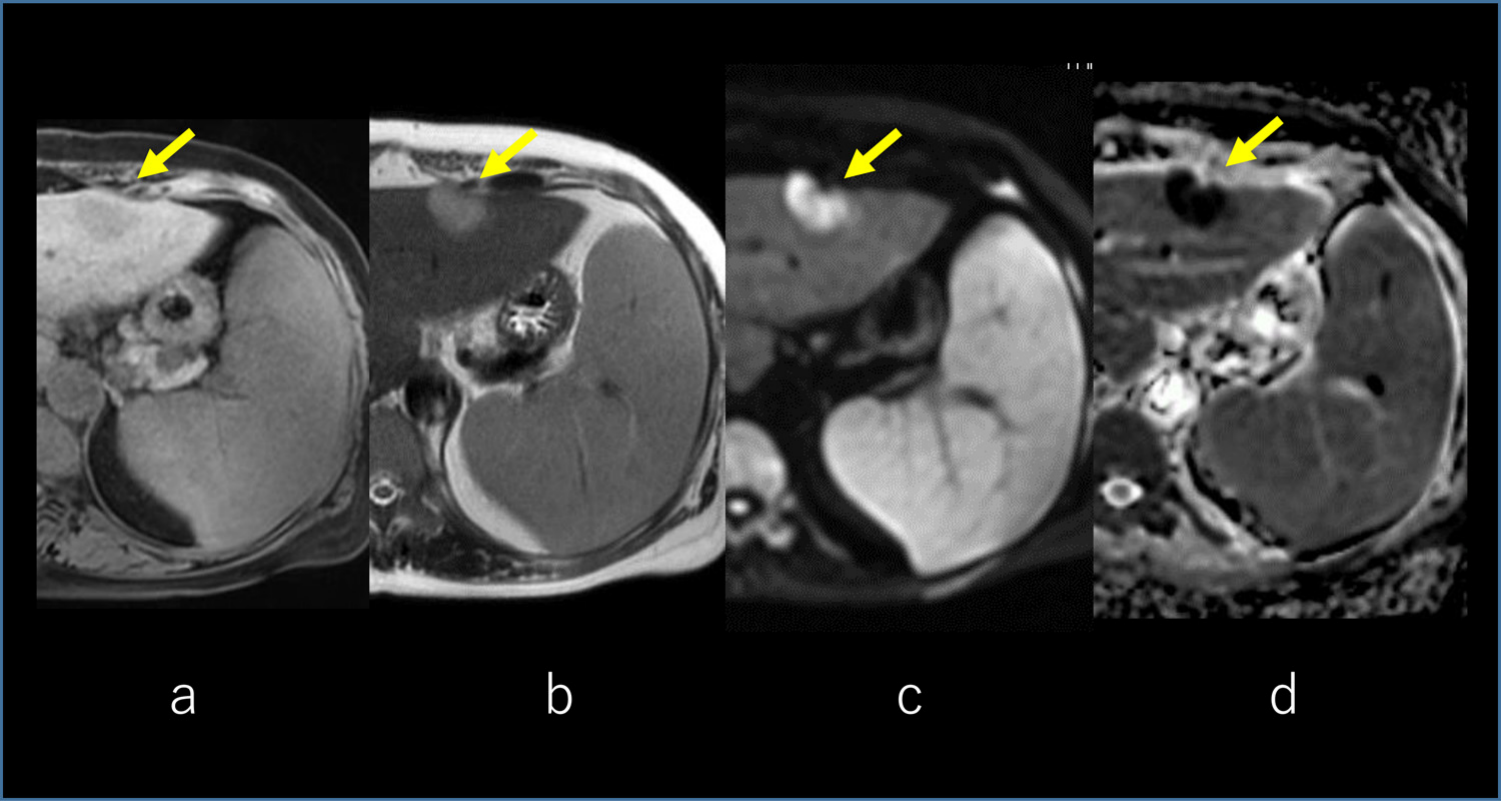
MRI of the liver. The tumor was visible in the left lobe which presents hypointense in T1-weighted image (arrow) **(A)** and slightly hyperintense in T2-weighted image (arrow) **(B)**. The lesion was presenting with a high signal in the diffusion-weighted image (DWI) (arrow) **(C)** and a low signal in the apparent diffusion coefficient (ADC) map (arrow) **(D)**. MRI, magnetic resonance imaging.

**Fig. 4:**
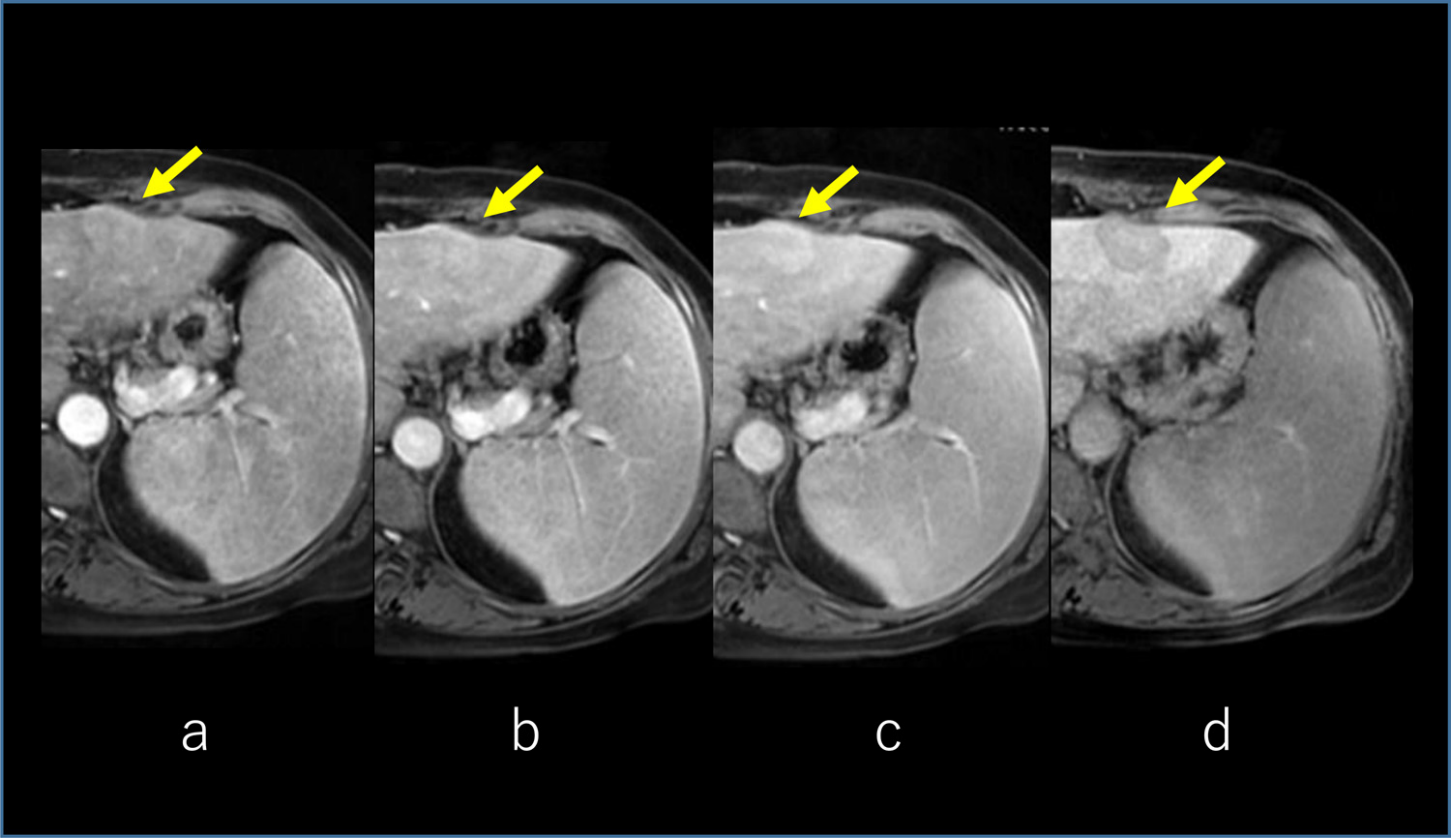
Gd-EOB-DTPA-enhanced MRI. Arterial, portal, and transitional phases of the dynamic scan show enhancement of the tumor with central part sparing (arrow) **(A, B, C)**. The hepatobiliary phase shows a faint uptake of contrast in the central part of the tumor (arrow) **(D)**. MRI, magnetic resonance imaging.

On magnetic resonance imaging (MRI) of the liver, the tumor was visible in the left lobe which presents hypointense in T1-weighted image (Fig. 3A) and slightly hyperintense in T2-weighted image (Fig. 3B). The lesion was presenting with a high signal in the diffusion-weighted image (Fig. 3C) and a low signal in the apparent diffusion coefficient map (Fig. 3D). Gadolinium-ethoxybenzyl-diethylenetriamine pentaacetic acid (Gd-EOB-DTPA)-enhanced MRI was performed for the liver lesion. Arterial, portal, and transitional phases of the dynamic scan showed enhancement of the tumor with central part sparing (Fig. 4A–C). The hepatobiliary phase showed a faint uptake of contrast in the central part of the tumor (Fig. 4D).

**Fig. 5:**
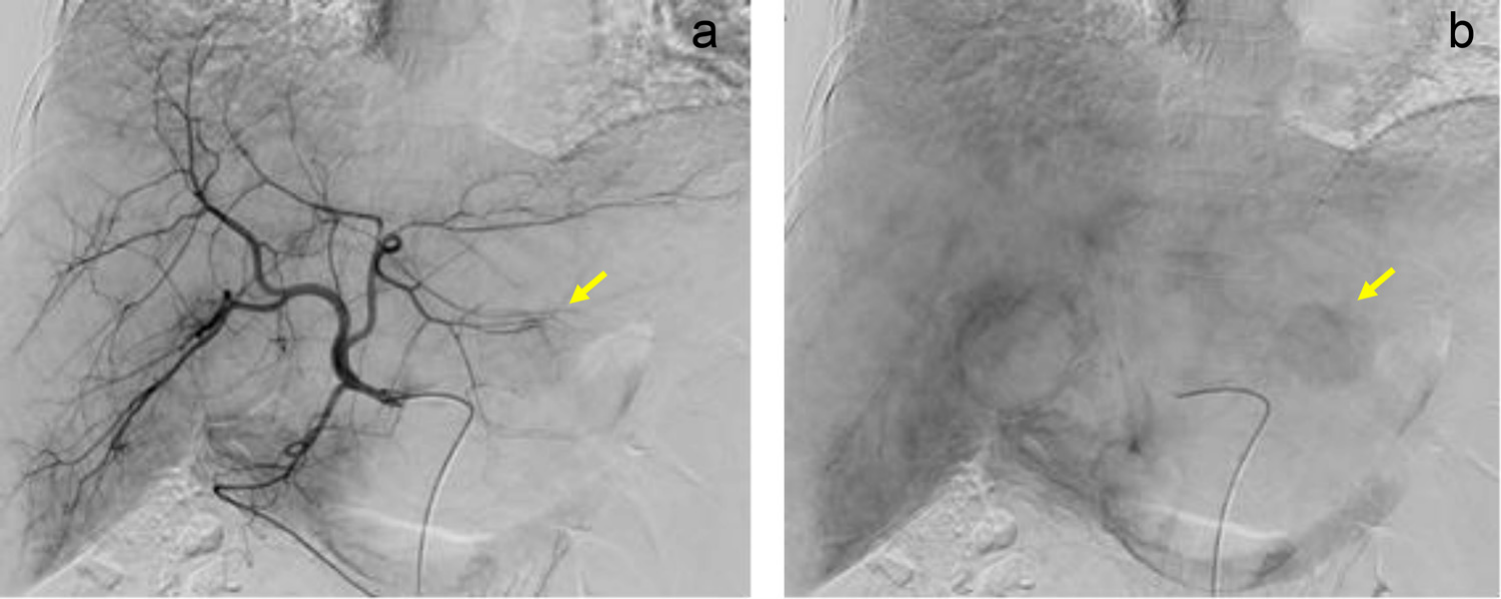
Hepatic arteriography. Early **(A)** and late **(B)** phases of hepatic arteriography show fine tumor staining (arrow) in segment 3.

Early and late phases of hepatic arteriography showed fine tumor staining in segment 3 (Fig. 5A and B). These were not typical findings for hepatocellular carcinoma or liver metastasis. Hepatic venous catheterization via the right arm was performed. The wedged hepatic venous pressure, free hepatic venous pressure, and hepatic venous pressure gradient were 24, 16 and 8 cmH2O, respectively. The result indicated mild portal hypertension. Endoscopy confirmed the absence of gastroesophageal varices.

**Fig. 6:**
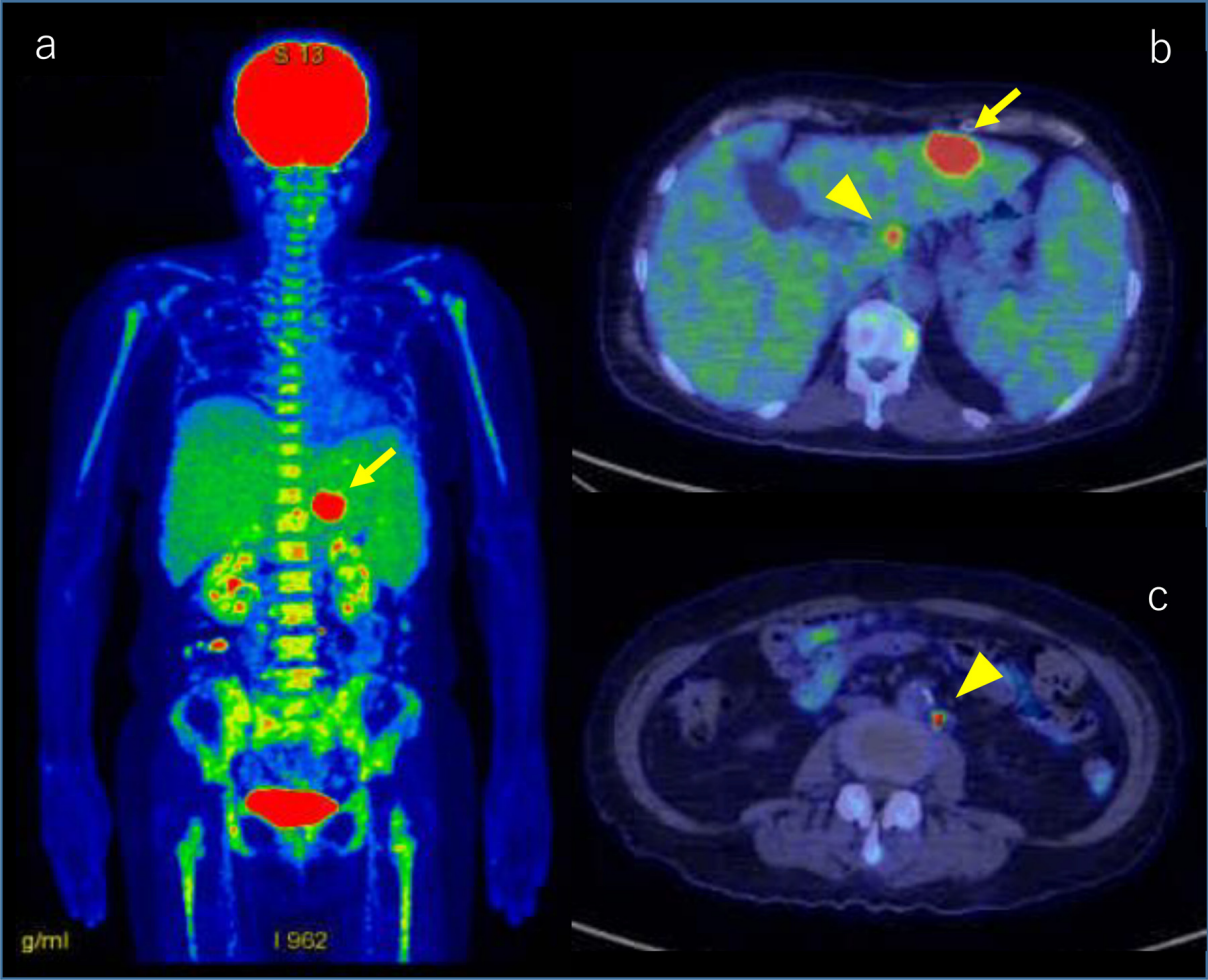
Whole-body scan of FDG-PET/CT. Coronal fusion image shows intensive uptake in the hepatic tumor (arrow), with an SUV max of 21.0 in segment 3 **(A)**. There is diffuse hyperaccumulation in the bone marrow of the spine, pelvic bones, ribs, and upper and lower limbs (SUV max, 5.6) **(A)**. Axial fusion image shows lymph nodes (arrowhead) around the hepatic artery **(B)** and around the aorta **(C)** with intensive uptake (SUV max, 21.9). FDG-PET/CT, fluorodeoxyglucose-positron emission tomography/computed tomography.

**Fig. 7:**
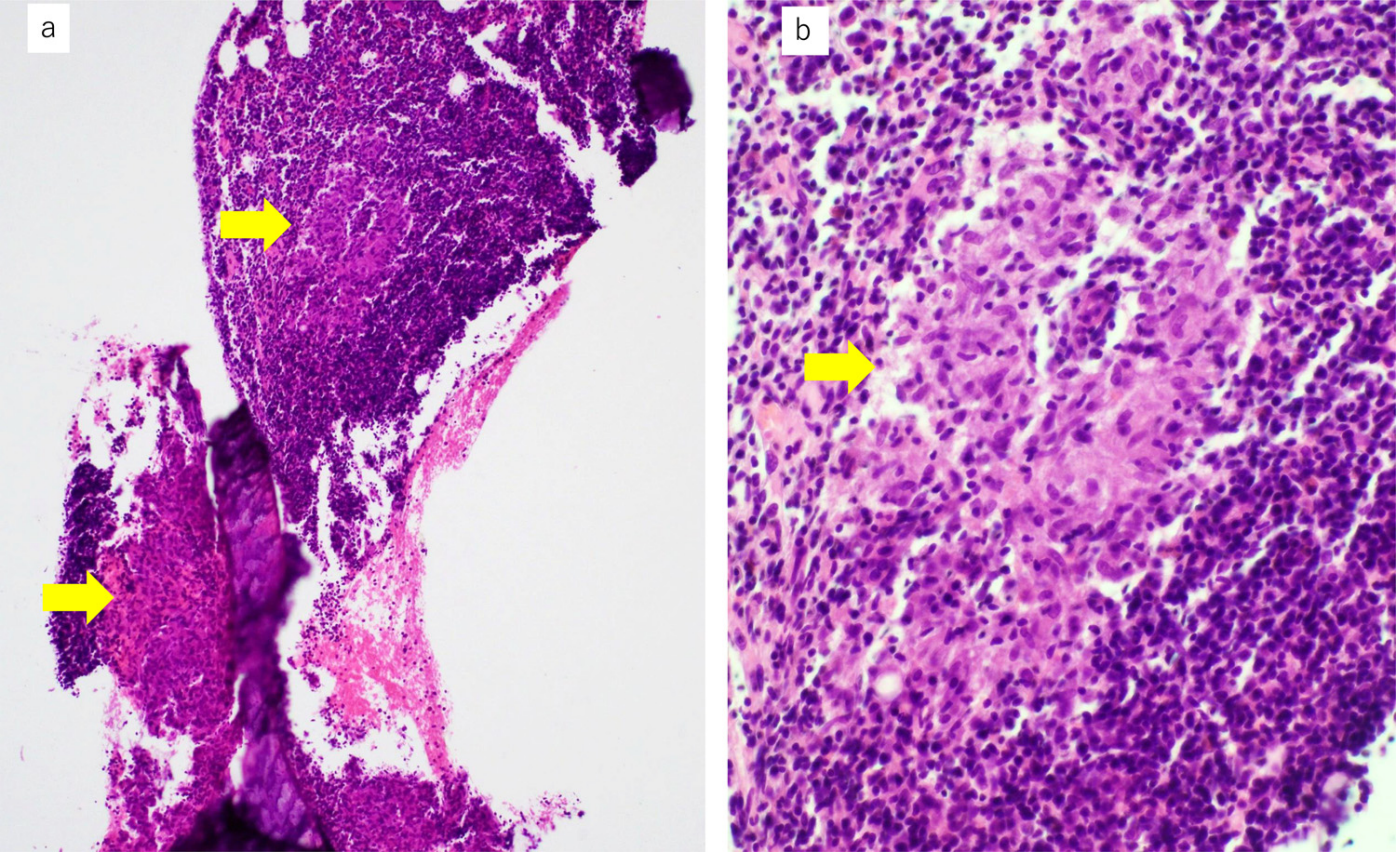
Histopathological findings of the biopsied bone marrow. The bone marrow shows multiple noncaseating epithelioid granulomas (**A**, lower magnification, ×40; **B**, higher magnification, ×400; granulomas indicated by arrows).

Whole-body scan of ^18^F-fluorodeoxyglucose (FDG)-positron emission tomography/computed tomography (PET/CT) was performed. In the liver, there were intensive uptakes in the tumor, with maximum standardized uptake values (SUV max) of 21.0 in segment 3. Accumulation of liver parenchyma other than the tumor was not abnormal. The accumulation of the spleen was equivalent to that of the liver, and it could be said that the accumulation was enhanced. There was diffuse hyperaccumulation in the bone marrow of the spine, pelvic bones, ribs, and upper and lower limbs (SUV max, 5.6). Axial fusion image showed lymph nodes (arrowhead) around the hepatic artery and around the aorta with intensive uptake (SUV max, 21.9) (Fig. 6A–C). Progressive malignant lymphoma was suspected on FDG-PET/CT, and bone marrow biopsy was performed. Histopathology showed multiple noncaseating epithelioid granulomas in the bone marrow (Fig. 7A and B). There was no histological evidence of lymphoma.

Laboratory investigations for possible causes of granulomas were added. Mycobacterium tuberculosis-specific interferon-γ release assay was negative. Angiotensin-converting enzyme was 29.3 IU/L/37 °C [7], [8], [9], [10], [11], [12], [13], [14], [15], [16], [17], [18], [19], [20], [21], [22], [24], [24], [25] and lysozyme was 18.3 µg/mL (5.0-10.0). These findings were consistent with sarcoidosis. However, no evidence of heart, lung, eyes, and skin sarcoid involvement was found. Ga scintigraphy showed no abnormal accumulation.

**Fig. 8:**
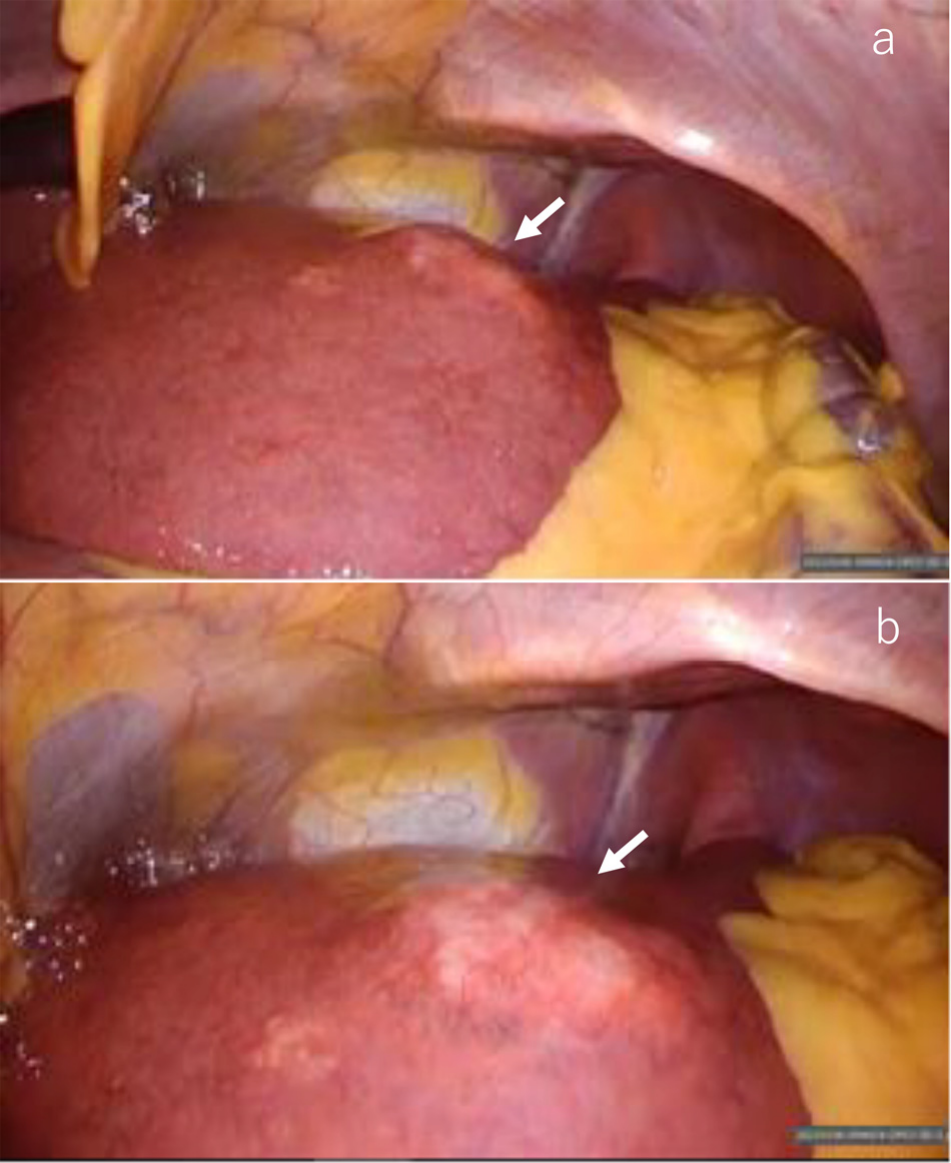
Laparoscopic view. Laparoscopic distal **(A)** and close-up views **(B)** show a slightly protruding white nodule (arrow).

**Fig. 9:**
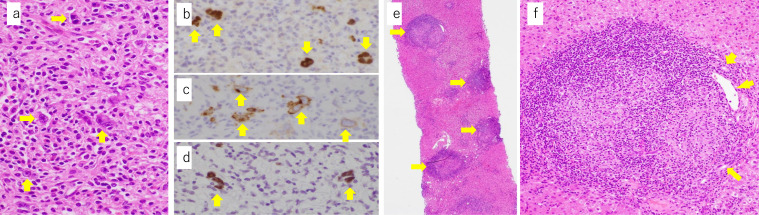
Histopathological findings of the liver biopsy. High magnification view shows scattered localization of Reed-Sternberg cells (**A**, ×400, arrows) which are immunohistochemically positive for PAX5 (**B**, ×400, arrows) and CD30 (**C**, ×400, arrows) and positive for Epstein-Barr virus-encoded small RNA 1 by in situ hybridization (**D**, ×400, arrows). The background hepatic parenchyma shows noncaseating epithelioid granulomas (arrows) developed mainly in the portal triads (**E**, ×40). The higher magnification view of the portal triad (**F**, ×200) including an epithelioid granuloma shows that the granuloma largely occupying the triad pushes bile ducts (arrows) and portal venule (arrowhead) to the periphery. The portal venule appears to be compressed by inflammation.

For histological diagnosis of the liver tumor and its extension, laparoscopic liver biopsy (Fig 8A and B) was selected over US-guided biopsy to control puncture hole bleeding due to portal hypertension and thrombocytopenia. Histological findings of the liver tumor demonstrated scattered localization of large atypical mononuclear and multinucleated lymphoid cells with distinct nucleoli embedded in many lymphocytes associated with plasma cells, eosinophils, and histiocytes. The large lymphoid cells were immunohistochemically positive for CD20, PAX5, CD15, and CD30, negative for CD3, and positive for Epstein-Barr virus-encoded small RNA 1 (EBER1) by in situ hybridization (Fig. 9A–D). The background lymphocytes mainly showed T lymphocytes and were negative for EBER1. Thus, classic Hodgkin lymphoma was highly suspected. There were multiple noncaseating epithelioid granulomas in the liver parenchyma, particularly in portal tracts (Fig. 9E–F). Portal venules in the portal tracts were inconspicuous probably because the epithelioid granulomas and lymphocytic infiltrates largely occupied the portal tracts (Fig. 9F). No NASH or bridging fibrosis was observed.

However, it was still unclear whether the splenomegaly was due to sarcoidosis, lymphoma, or any other causes. Therefore, splenectomy for splenomegaly and partial hepatectomy for liver tumor were performed. Intraoperative portal venous pressure decreased from 24.5 to 13.5 cmH2O immediately after splenectomy. There were gallbladder stones, and cholecystectomy was performed at the same time.

**Fig. 10:**
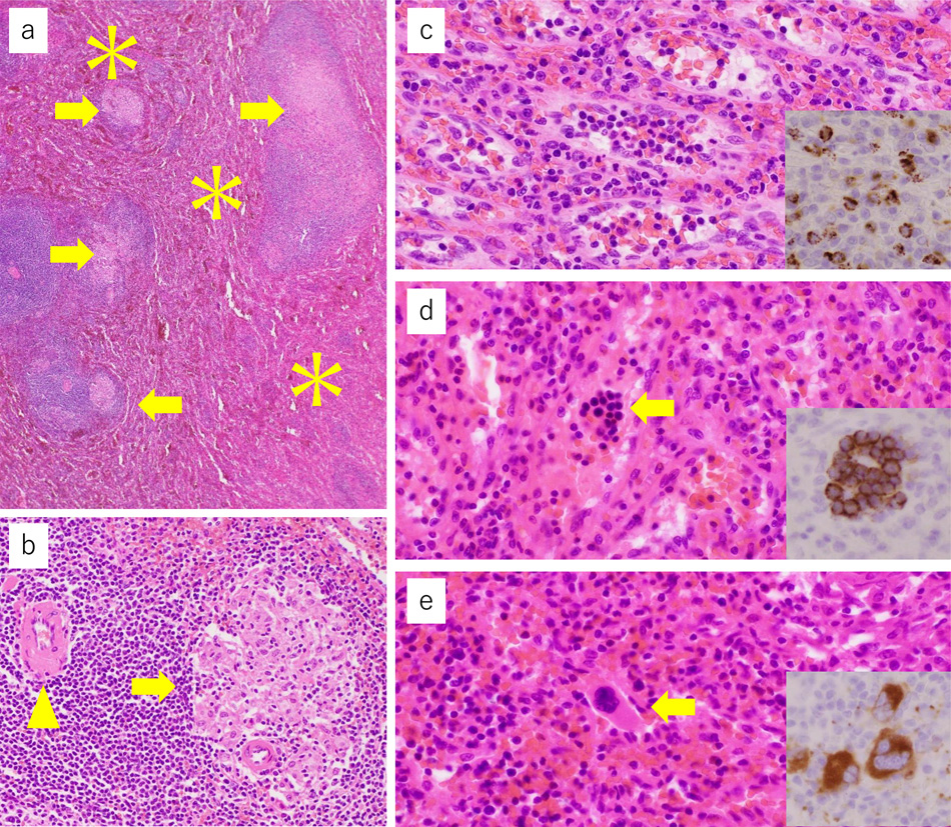
Histopathological findings of the resected spleen. The spleen pathologically shows that multiple noncaseating epithelioid granulomas (**A, B**, arrows) are mainly present in the white pulps (**B**, a splenic central artery is indicated by an arrowhead) and red pulps (asterisks) are extended (**A**, ×40). Red pulps demonstrate extramedullary hematopoiesis because, in red pulps, there are neutrophils (**C**, ×400) and myeloperoxidase-immunoreactive myeloid cells (**C**, inset, ×400), erythroblastic islands (**D**, ×400) that are immunohistochemically positive for CD71 (**D**, inset, ×400), and megakaryocytes (**E**, ×400) that are immunohistochemically positive for CD61 (**E**, inset, ×400).

Two weeks after surgery, sIL-2R, angiotensin-converting enzyme, and lysozyme were reduced to 2226 U/mL, 16.5 IU/L/37 °C, and 12.1µg/mL, respectively. Platelet count increased to 25.8 × 10^4^ /µL. Pathological examination of the resected specimens revealed multiple noncaseating epithelioid granulomas in the liver, spleen, gallbladder, and small lymph nodes near the cystic duct. In the liver and spleen, most noncaseating epithelioid granulomas were present in hepatic portal tracts and splenic white pulps (Fig. 10A and B). Portal venules in the portal tracts were inconspicuous as the epithelioid granulomas associated with lymphocytic infiltrates largely occupied the portal tracts. The liver tumor was pathologically confirmed as classic Hodgkin lymphoma with mixed cellularity. Hepatic steatosis was absent and mild fibrosis was associated around granulomas; however, neither bridging fibrosis nor cirrhosis was found. In the spleen, increased areas of red pulps were present (Fig. 10A), where extramedullary hematopoiesis was observed (Fig. 10C–E). No breast cancer metastasis was found in the resected tissues.

**Fig. 11:**
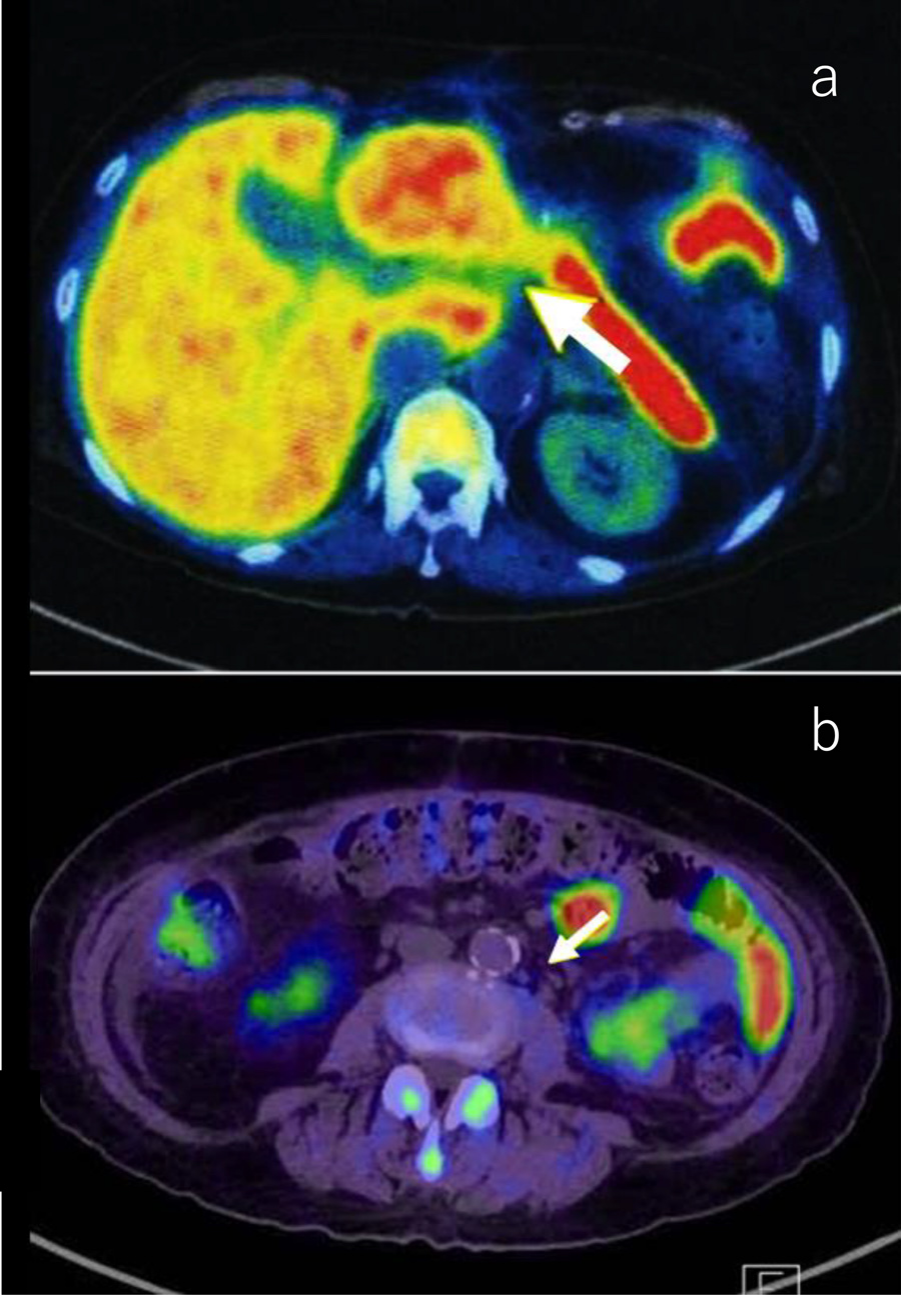
Axial fusion image of MET-PET/CT after the surgery. Lymph nodes around the hepatic artery **(A)** and around the aorta **(B)**, which showed high accumulation on FDP-PET/CT, show low accumulation on MET-PET/CT (SUV max, 3.7 and 1.3, respectively). The findings are consistent with sarcoidosis. MET-PET/CT, methionine-positron emission tomography/computed tomography.

^11^C-methionine (MET)-PET/CT was performed after the surgery. Lymph nodes around the hepatic artery and around the aorta, which showed high accumulation by FDP-PET/CT, showed low accumulation by MET-PET/CT. The findings were consistent with sarcoidosis (Fig. 11A and B). From the above results, we were able to make a final diagnosis of primary hepatic Hodgkin lymphoma and sarcoidosis (sarcoidosis-lymphoma syndrome) with portal hypertension. The patient is scheduled for adjuvant chemotherapy [11,12] in the near future.

## Discussion

Sarcoidosis is a multisystem disorder of unknown origin. The diagnosis is established when clinicopathological findings are supported by pathological evidence of non-caseating epithelioid granulomas. Many immunologic abnormalities were observed in patients with sarcoidosis [13]. Although granulomas are found in as many as 50%-80% of liver biopsy, hepatic sarcoidosis with portal hypertension is rare [7], [8], [9], and sarcoidosis-lymphoma syndrome with portal hypertension is rarer. Its diagnostic approach has not been established. We report the usefulness of hepatic venous catheterization, PET/CT, and pathological examinations of bone marrow, liver, spleen, and lymph nodes for the diagnosis of sarcoidosis-lymphoma syndrome with or without portal hypertension.

Seventeen cases of sarcoidosis-lymphoma syndrome were reported in 1986 [1]. In their series, hepatosplenomegaly was found only in 1 case and bone lesions were found in only 2 cases. There were 8 cases of Hodgkin disease, 4 of non-Hodgkin disease, 3 of chronic lymphocytic leukemia, and 2 of paraproteinemia. Furthermore, he found additional malignant tumors in 5 patients. Our case also had a history of breast cancer 2 years ago. He reported the mean interval from sarcoidosis to malignant lymphoproliferative disease was 81 months for Hodgkin disease, and 89 months for non-Hodgkin disease. In our case, it is possible that sarcoidosis was a primary driver for hepatosplenomegaly and hypersplenism that was noted 2 years ago.

**Fig. 12:**
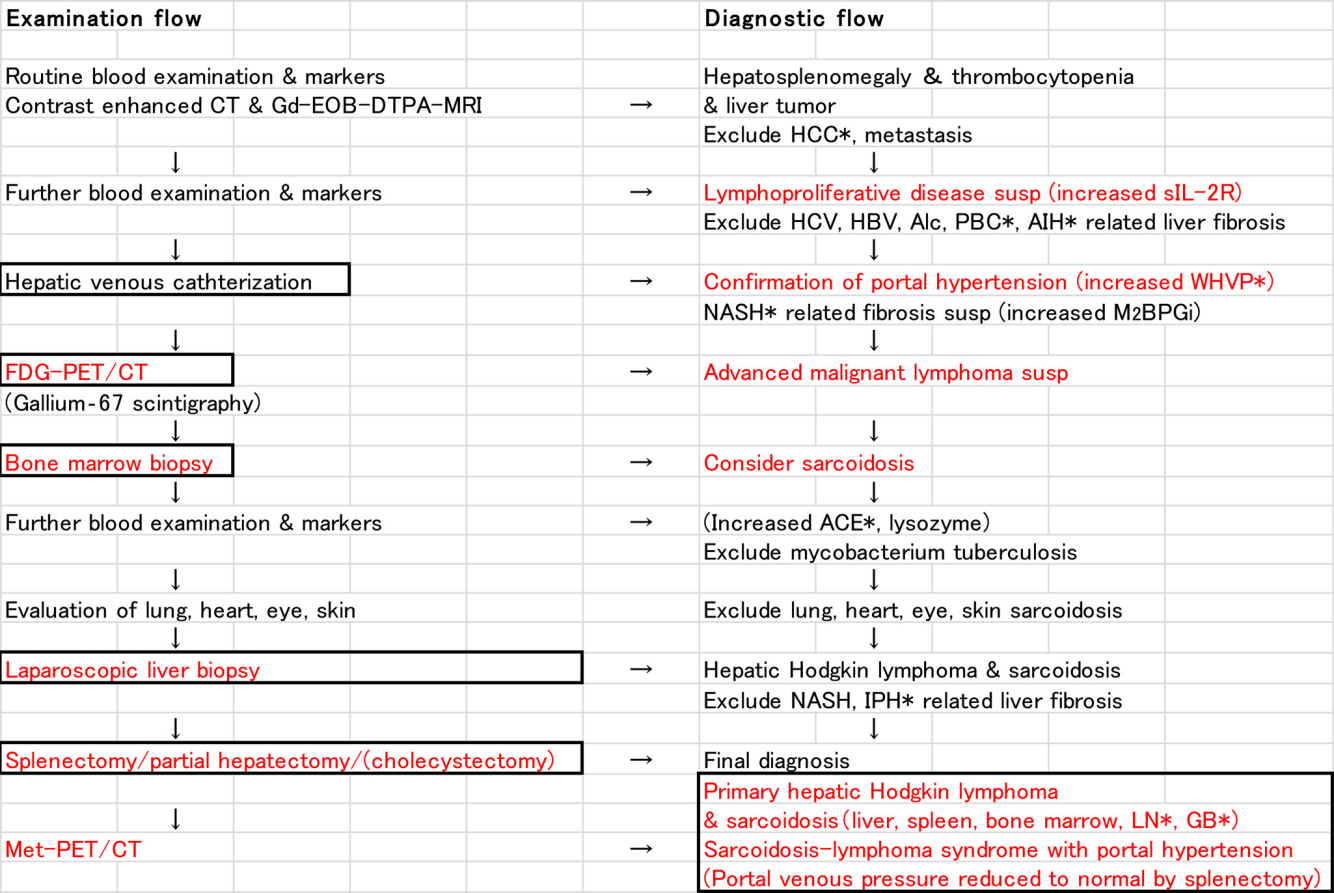
Examination and diagnostic flow of the case. HCC, hepatocellular carcinoma; PBC, primary biliary cirrhosis; AIH, autoimmune hepatitis; WHVP, wedged hepatic venous pressure; ACE, angiotensin-converting enzyme; IPH, idiopathic portal hypertension; LN, lymphnodes; GB, gallbladder.

The flow of examination and diagnosis of this case is shown in Figure 12. M2BPGi is a serum biomarker for assessing liver fibrosis. Liver fibrosis is the most common cause of portal hypertension. Because M2BPGi was increasing, we initially suspected NASH-related liver fibrosis as the primary cause of splenomegaly. However, the pathological examination did not show NASH-related fibrosis. Because M2BPGi increases in patients with acute liver injury, this marker seems to reflect not only liver fibrosis but also other factors, such as liver inflammation, liver damage, and hepatocyte regeneration [14], [15], [16]. The relationship between M2BPGi and sarcoidosis seems to be an issue for future studies.

The imaging manifestations of hepatic lymphoma are largely non-specific [17]. Higuma et al. [18] reported a case of hepatic Hodgkin lymphoma with delayed enhancement on CT and MRI. Our case also had a hepatic tumor with delayed enhancement on CT, MRI, and hepatic arteriography, however, we only vaguely suspected malignant lymphoma with reference to increased sIL-2R.

FDG-PET/CT is the basis for searching for malignant diseases. Progressive malignant lymphoma was suspected on FDG-PET/CT (Fig. 6A and B); however, bone marrow biopsy showed multiple noncaseating granulomas (Fig. 7A and B). At this point, we considered the presence of sarcoidosis. The diagnostic ability of FDG-PET/CT is higher than Ga scintigraphy for detection of extrapulmonary granulomas [19]. Higher FDG-uptake is seen in both sarcoidosis and malignancy. Therefore, FDG-PET/CT cannot exclude or prove the presence of malignancy in patients with sarcoidosis. The histopathological study is necessary to confirm the diagnosis. Laparoscopic liver biopsy of the tumor confirmed the diagnosis of Hodgkin lymphoma (Fig. 9A and B). Multiple granulomas in the parenchyma of the liver, and no NASH or bridging fibrosis was observed (Fig. 9C). However, it was still unclear whether the splenomegaly was due to sarcoidosis, lymphoma, or any other causes. Curative hepatic resection with adjuvant chemotherapy for primary hepatic lymphoma was considered as adequate treatment [20]. Therefore, splenectomy for splenomegaly and partial hepatectomy for liver tumor were performed.

Recently, the use of amino acid analog radiotracers, such as ^11^C-methionine (MET) and ^18^F-α-methyltyrosine (FAMT) in malignancy has been developed and clinically established. Accumulation of amino acid radiotracers in malignant tissues is thought to be due to increased amino acid metabolism. Several clinical trials on the evaluation of sarcoid lymphadenopathy using MET and FAMT in comparison with FDG have been published [21], [22], [24]. It is likely that high FDG accumulation in sarcoidosis lesions is due to the abundant presence of inflammatory cells and granulomas. On the other hand, the accumulation of MET is considered to be low because MET uptake in inflammation is low. In our case, MET-PET/CT was performed after partial hepatectomy and splenectomy. From findings of low accumulation of MET in lymphadenopathies (Fig. 11A and B), a final diagnosis of primary hepatic Hodgkin lymphoma with sarcoidosis was established.

Hepatic venous catheterization is a basic examination for portal hypertension. Use of hepatic venous catheterization for the assessment of hepatic sarcoidosis has also been reported [7,8]. Although this case was not accompanied by the portal collateral development, we confirmed an increased wedged hepatic venous pressure. The pathophysiology of portal hypertension in sarcoidosis is not completely understood. Multiple mechanisms, such as small arterio-venous shunts in the region of the granulomas, healing fibrosis of the parenchyma, cirrhotic remodeling, and restriction of normal flow by granulomas in the portal area have been suggested [24], [25], [26], [27]. In our case, it was proved that the cause of portal hypertension was mainly increased splenic venous blood flow volume associated with marked splenomegaly (Figs 2 and 10A and B). Disturbed portal circulation by hepatic granulomas mainly developed in the portal triads was also involved in the cause (Fig. 10C).

It is estimated that 7% of patients with sarcoidosis have splenic involvement, which is commonly asymptomatic [2]. Managements of hepatic sarcoidosis with splenomegaly have not also been established. Splenectomy is often recommended for hypersplenism after medical management. We have reported that partial splenic embolization can reduce the splenic venous blood flow volume and portal venous pressure [28] in cirrhotic patients. However, in this case, we applied splenectomy instead of partial splenic embolization because pathological examination of the spleen was necessary to prove whether the splenomegaly was due to sarcoidosis, lymphoma, or any other causes. The management of splenomegaly is also important in the treatment of portal hypertension based on a new concept of “Splanchnic Caput Medusae” in which the enlarged spleen is her face and portal collateral pathways are her snake hairs [29]. In this case, portal venous pressure was dramatically reduced to a normal level immediately after splenectomy.

Sarcoidosis-lymphoma syndrome is rare, hepatic sarcoidosis with portal hypertension is uncommon, and primary hepatic Hodgkin lymphoma is infrequent. Our case of primary hepatic Hodgkin lymphoma with sarcoidosis accompanied by portal hypertension is extremely rare. The diagnosis was difficult. Two things could be learned from the current case. One was the diagnostic approach and the other was the pathophysiology. We conclude that hepatic venous catheterization, PET/CT, and pathological examinations of bone marrow, liver, spleen, and lymph nodes are crucial for the diagnosis of primary hepatic Hodgkin lymphoma with sarcoidosis (sarcoidosis-lymphoma syndrome) accompanied by portal hypertension.

## Patient consent statement

Written informed consent was obtained from the patient for publication of this case report and accompanying images.

## References

[1] Brincker H. (1986). The sarcoidosis-lymphoma syndrome. Br J Cancer.

[2] Papanikolaou IC, Sharma OP. (2010). The relationship between sarcoidosis and lymphoma. Eur Respir J.

[3] Oskuei A, Hicks L, Ghaffar H, Hoffstein V. (2017). Sarcoidosis-lymphoma syndrome: a diagnostic dilemma. BMJ Case Rep.

[4] Yamanaka T, Kanai H, Aihara N, Ohno T, Mase M. (2019). A case of sarcoidosis-lymphoma syndrome: importance of brain biopsy. NMC Case Report J.

[5] Kis A, Eszes N, Tamasi L, Losonczy G, Csekeo A, Csomor J (2013). Sarcoidosis lymphoma syndrome—the value of PET-CT in the diagnosis. World J Sur Oncol.

[6] Di L, Wang CP, Tang J, Macaulay R, Tran N. (2021). Sarcoidosis-lymphoma syndrome presenting as bony vertebral metastasis: a case report and literature review. Cureus.

[7] Maddrey WC, Johns CJ, Boitnott JK, Iber FL. (1970). Sarcoidosis and chronic hepatic disease: a clinical and pathologic study of 20 patients. Medicine.

[8] Mistillis SP, Green JR, Schir L. (1964). Hepatic sarcoidosis with portal hypertension. Am J Med..

[9] Rosenberg JC. (1971). Portal hypertension complicating hepatic sarcoidosis. Surgery.

[10] Chikamori F, Nishida S, Selvaggi G, Tryphonopoulos P, Moon JI, Levi DM (2010). Effect of liver transplantation on spleen size, collateral veins, and platelet counts. World J Surg.

[11] Behringer K, Goergen H, Hitz F, Zijlstra JM, Greil R, Markova J (2015). Omission of dacarbazine or bleomycin, or both, from the ABVD regimen in treatment of early-stage favourable Hodgkin's lymphoma (GHSG HD13): an open-label, randomised, non-inferiority trial. Lancet.

[12] Engert A, Plütschow A, Eich HT, Lohri A, Dörken B, Borchmann P (2010). Reduced treatment intensity in patients with early-stage Hodgkin's lymphoma. N Engl J Med.

[13] (1999). Statement on sarcoidosis. Joint Statement of the American Thoracic Society (ATS), the European Respiratory Society (ERS) and the World Association of Sarcoidosis and Other Granulomatous Disorders (WASOG) adopted by the ATS Board of Directors and by the ERS Executive Committee, February 1999. Am J Respir Crit Care Med.

[14] Uchiyama H, Shirabe K, Bekki Y, Toshima T, Harimoto N, Ikegami T (2019). Peritransplant kinetics of Mac-2-binding protein glycosylation isomer levels in living donor liver transplantation: its implication of posttransplant small-for-size syndrome. Transl Gastroenterol Hepatol.

[15] Morio K, Imamura M, Daijo K, Teraoka Y, Honda F, Nakamura Y (2017). Wisteria floribunda agglutinin positive Mac-2-binding protein level increases in patients with acute liver injury. J Gastroenterol.

[16] Yamada N, Katano T, Hirata Y, Okada N, Sanada Y, Ihara Y (2019). Serum Mac-2 binding protein glycosylation isomer predicts the activation of hepatic stellate cells after liver transplantation. J Gastroenterol Hepatol.

[17] Rajesh S, Bansal K, Sureka B, Patidar Y, Bihari C, Arora A. (2015). The imaging conundrum of hepatic lymphoma revisited. Insights Imag.

[18] Higuma Y, Yamauchi R, Fujimitsu R, Sakamoto K, Shinagawa Y, Morita A, et al.Hepatic Hodgkin lymphoma with delayed enhancement on CT and MRI Radiol Case Rep. 2016; 12(1): 45-9. doi: 10.1016/j.radcr.2016.11.013. 10.1016/j.radcr.2016.11.013 PMC5310377 28228877

[19] Nishiyama Y, Yamamoto Y, Fukunaga K, Takinami H, Iwado Y, Satoh K (2006). Comparative evaluation of ^18^F-FDG PET and ^67^Ga scintigraphy in patients with sarcoidosis. J Nucl Med.

[20] Taketomi A, Takenaka K, Shirabe K, Matsumata T, Maeda T, Shimada M (1996). Surgically resected primary malignant lymphoma of the liver. Hepatogastroenterology.

[21] Hoffman RM. (2019). L-[Methyl-(11)C] Methionine-Positron-Emission Tomography (MET-PET). Methods Mol Biol.

[22] Yudistiro R, Arisaka Y, Tokue A, Nakajima T. (2016). Differentiation of sarcoidosis-lymphoma syndrome lesions: a case report on the use of two different positron emission tomography tracers. BMC Med Imag.

[24] Yamada Y, Uchida Y, Tatsumi K, Yamaguchi T, Kimura H, Kitahara H (1998). Fluorine-18-fluorodeoxyglucose and carbon-11-methionine evaluation of lymphadenopathy in sarcoidosis. J Nucl Med..

[24] Blich M, Edoute Y (2000). Clinical manifestations of sarcoid liver disease. J Gastroenterol Hepatol.

[25] Ebert EC, Kierson M, Hagspiel KD. (2008). Gastrointestinal and hepatic manifestations of sarcoidosis. Am J Gastroenterol.

[26] Tan CB, Rashid S, Rajan D, Gebre W, Mustacchia P. (2012). Hepatic sarcoidosis presenting as portal hypertension and liver cirrhosis: case report and review of the literature. Case Rep Gastroenterol.

[27] Saito S, Kodama K, Kogiso T, Yamanashi Y, Taniai M, Ariizumi S (2020). Atypical sarcoidosis diagnosed by massive splenomegaly. Intern Med.

[28] Chikamori F, Kuniyoshi N, Kawashima T, Takase Y. (2007). Short-term portal hemodynamic effects of partial splenic embolization for hypersplenism. Hepatogastroenterology.

[29] Chikamori F, Sharma N, Ito S, Mizobuchi K, Ueta K, Takasugi H (2020). Stepwise partial splenic embolization for portal hypertension based on a new concept: splanchnic caput Medusae. Radiol Case Rep.

